# Sialylated immunoglobulin G: a promising diagnostic and therapeutic strategy for autoimmune diseases

**DOI:** 10.7150/thno.53961

**Published:** 2021-03-13

**Authors:** Danqi Li, Yuchen Lou, Yamin Zhang, Si Liu, Jun Li, Juan Tao

**Affiliations:** 1 Department of Dermatology, Union Hospital, Tongji Medical College, Huazhong University of Science and Technology, Wuhan, China.; 2 Hubei Engineering Research Center for Skin Repair and Theranostics, Wuhan, China.; 3 Britton Chance Center for Biomedical Photonics at Wuhan National Laboratory for Optoelectronics-Hubei Bioinformatics & Molecular Imaging Key Laboratory, Systems Biology Theme, Department of Biomedical Engineering, College of Life Science and Technology, Huazhong University of Science and Technology, Wuhan, China.; 4 Department of Dermatology, The Central Hospital of Wuhan, Tongji Medical College, Huazhong University of Science and Technology (HUST), Wuhan, China.

**Keywords:** sialylation, immunoglobulin G, glycosylation, autoimmune diseases, precision medicine

## Abstract

Human immunoglobulin G (IgG), especially autoantibodies, has major implications for the diagnosis and management of a wide range of autoimmune diseases. However, some healthy individuals also have autoantibodies, while a portion of patients with autoimmune diseases test negative for serologic autoantibodies. Recent advances in glycomics have shown that IgG Fc *N*-glycosylations are more reliable diagnostic and monitoring biomarkers than total IgG autoantibodies in a wide variety of autoimmune diseases. Furthermore, these *N*-glycosylations of IgG Fc, particularly sialylation, have been reported to exert significant anti-inflammatory effects by upregulating inhibitory FcγRIIb on effector macrophages and reducing the affinity of IgG for either complement protein or activating Fc gamma receptors. Therefore, sialylated IgG is a potential therapeutic strategy for attenuating pathogenic autoimmunity. IgG sialylation-based therapies for autoimmune diseases generated through genetic, metabolic or chemoenzymatic modifications have made some advances in both preclinical studies and clinical trials.

## Introduction

Autoimmune diseases are a heterogeneous group of debilitating and painful conditions characterized by immune dysregulation resulting in inflammation and multiorgan involvement 1-3. The presence of pathogenic autoantibodies is one of the characteristics of autoimmune diseases and is usually regarded as a diagnostic biomarker 4,5. In addition to early diagnosis, these biomarkers could provide clinicians with useful information about risk stratification, therapeutic efficacy, and prognosis. However, it has been reported that up to 20-30% of the healthy population is positive for antinuclear antibodies 6. Moreover, patients with certain conditions, such as laboratory sine syndromes and anti-citrullinated protein antibody (ACPA)-negative rheumatoid arthritis (RA), are negative for commonly specific autoantibodies 7. For example, 15-25% of RA patients are negative for rheumatoid factor (RF) and ACPA 8,9.

In addition, although the advent and development of biologic agents, especially IgG-type monoclonal antibodies (mAbs), marks an unparalleled opportunity to advance therapeutic strategies for rheumatic diseases, some of these recombinant antibodies might aggravate the inflammatory response or induce new onset of pathological conditions. The exacerbation or new onset of psoriasis-like skin lesions is a common adverse event associated with TNF-α inhibitors 10,11. Nearly 2-5% of patients with Crohn's disease and RA receiving anti-TNF-α mAbs (infliximab, adalimumab and etanercept) were associated with an increased risk of paradoxical psoriasis due to prolonged type I interferon production by immature plasmacytoid dendritic cells (pDCs) in the dermis 10,12. Thus, there is an urgent need for the identification of more precise diagnostic biomarkers with higher sensitivity and specificity as well as more effective therapeutic strategies with fewer proinflammatory adverse events based on IgG-type autoantibodies and mAbs.

**Figure 1:**
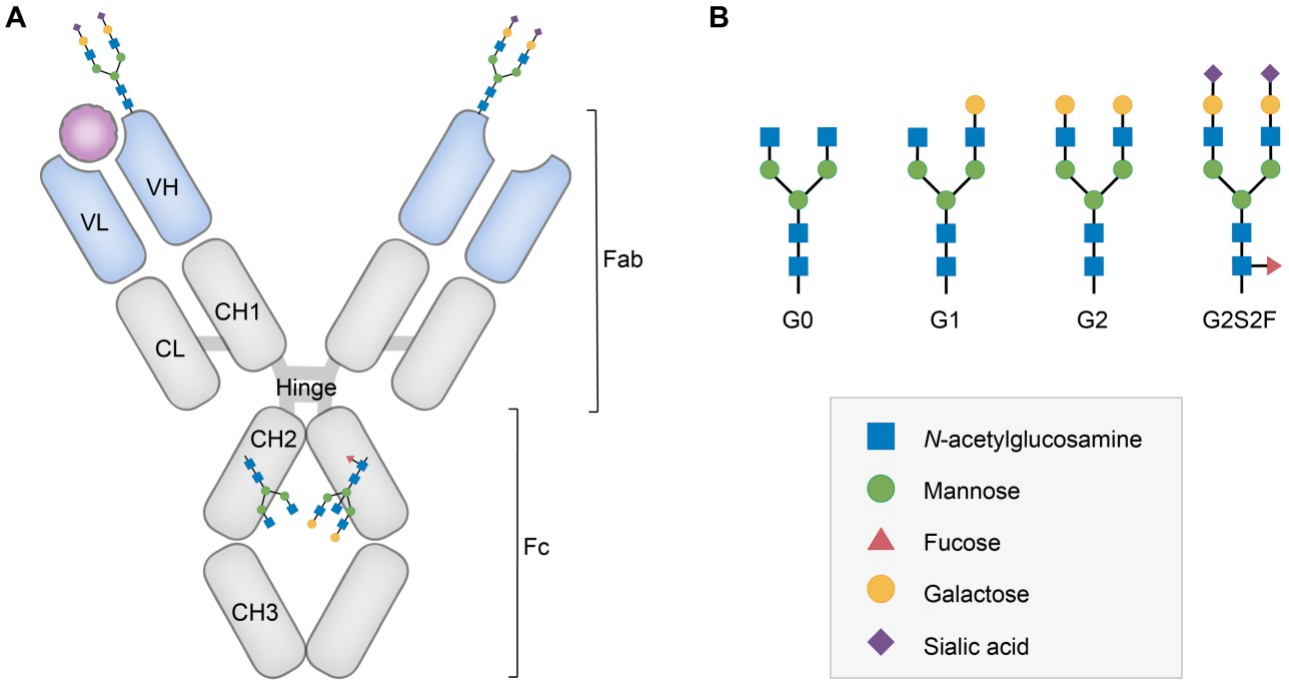
** Structure and glycan composition of IgG. A.** Schematic representation of immunoglobulin G with heavy chains and light chains in a general Y-shaped structure. Fab glycans are usually more galactosylated and sialylated but less fucosylated than Fc *N*-glycans. Fc glycans are rarely fully processed and can differ in composition between each CH2 domain of the same IgG molecule. **B.** The classification of Fc Asn-297 glycans by different glycoforms. *N*-glycans can also be galactosylated, sialylated or fucosylated, and these modifications alter the anti-inflammatory activity of IgG. The anti-inflammatory activity of immunoglobulin with different Fc *N*-glycan modifications increases from G0 to G2S2F. Ig, immunoglobulin; CH, constant heavy; CL, constant light; VH, variable heavy; VL, variable light; G, galactose; S, sialic acid; F, fucose.

Glycosylation fingerprints are now considered critical regulators to fine-tune the steric conformation and function of IgG subclasses. All IgG molecules contain a biantennary oligosaccharide attached to the conserved *N*-linked glycosylation site (Asn-297) of each Fc-CH2 domain 13,14. This glycan possesses a conserved heptamer consisting of *N*-acetylglucosamine (GlcNAc) and mannose residues, which can be further decorated by fucose, bisecting GlcNAc, galactose and sialic acid (Figure 1) 15-17. Since the 1980s, mounting evidence has favored the presence of unique glycosylation patterns of IgG (mainly sialylation, fucosylation and galactosylation) in autoimmune diseases 18. It is noteworthy that certain types of glycosylations of IgG, particularly sialylated autoantibodies, can help accurately diagnose antiphospholipid syndrome (APS) and RA 9,19. In addition, decreased levels of IgG sialylation were observed in samples from RA patients compared to arthralgia samples and may predict therapy resistance in Kawasaki disease 20,21. Furthermore, sialylated intravenous immunoglobulin (IVIg) and autoantibodies lead to significantly reduced joint inflammation or cytotoxicity of anti-platelet antibodies in RA or immune thrombocytopenic purpura (ITP) compared to those associated with their nonglycosylated counterparts 22. Defucosylated anti-C-C chemokine receptor 4 (CCR4) IgG1 has been approved by the US Food and Drug Administration (FDA) for the treatment of relapsed or refractory mycosis fungoides or Sézary syndrome 23,24. The presence of the above glycans further dictates specific Fc-Fc gamma receptor (FcγR) interactions, thus governing complement and immune cell activation in different ways 25,26. Reduced core fucosylation was found in SLE, which resulted in an enhanced capacity to activate natural killer (NK) cells and macrophages 27,28. In contrast, binding of the terminal sialic acid on IgG Fc to type II FcγRs can suppress proinflammatory effects, such as B cell receptor (BCR) signaling, by increasing inhibitory FcγRIIb expression 29. Furthermore, the significant role played by defucosylation in promoting antitumor mAbs in antibody-dependent cellular cytotoxicity (ADCC) has been extensively demonstrated, while sialylated autoantibodies and/or IgG are a promising strategy during the process of diagnosing and treating autoimmune diseases because of their anti-inflammatory effect 30.

In this review, we summarize the influence of IgG Fc *N*-linked glycan in autoimmune diseases, focusing on recent advances in understanding the role of sialylated autoantibodies and IgG in clinical diagnosis and treatment. We also touch upon current glycoengineering approaches used in this area and future perspectives.

## IgG Fc and FcγRs

### Structure and function of IgG Fc and FcγRs

The antigen-binding fragment (Fab) regions of IgG dictate target specificity, while the crystallizable fragment (Fc) regions contain interaction sites for sensors that elicit clearance, destruction or immunomodulatory signals 31. Based on their binding stoichiometry and recognized Fc sites, FcγRs are mainly divided into types I and II, with distinct expression patterns across cell subsets and tissues 32. Type I FcγRs fall within the immunoglobulin receptor superfamily and are classified as activating or inhibitory FcγRs on the basis of their intracellular domain signaling motifs 13,33. Activating type I FcγRs bind Fc near its hinge-proximal region, driving proinflammatory signaling pathways 34,35. The downstream effects of activating type I FcγRs include ADCC, antibody-dependent cellular phagocytosis (ADCP), leukocyte activation and proinflammatory cytokine release 15. It is worth mentioning that FcγRIIb is the only inhibitory receptor among type I FcγRs, being able to antagonize and balance signals of activating FcγRI or BCR 31. XmAb5871 is an engineered humanized anti-CD19 mAb that can also bind to FcγRIIb with 400-fold higher affinity than native IgG1 via its modified Fc. Thus, XmAb587 can suppress BCR signaling in B cells by promoting the coengagement of FcγRIIb with CD19. To date, it has realized promising therapeutic efficacy in a phase 2 clinical trial recruiting patients with IgG4-related diseases (NCT02725476) 36,37. Of note, each FcγR has a unique pattern of cellular expression. For example, B cells express inhibitory FcγRIIb as their sole type I FcγRs, whereas NK cells exclusively express activating FcγRs (FcγRIIIa) 38. FcγRIIIa on NK cells contributes to ADCC, while the upregulation of FcγRIIb on effector macrophages is achieved by sialylated IVIg 34. Most other immune cells express a combination of diverse FcγRs, pairing activating and inhibitory receptors to trigger specific effector functions.

Type II FcγRs belong to the C-type lectin receptor family and have an extracellular oligomeric structure to bind Fc at the CH2-CH3 interface 34,35. Dendritic cell-specific ICAM-3-grabbing nonintegrin (DC-SIGN, SIGN-R1 in mice) and CD23 are representative type II FcγRs. SIGN-R1 is expressed mainly on macrophages in mice, whereas DC-SIGN is expressed primarily by monocytes, dendritic cells and macrophages in humans 39. CD23 exists in two splice variants, CD23a and CD23b, with their first seven amino acid residues differing. The former is constitutively expressed by mature B cells, whereas the latter is only expressed in association with IL-4 on a variety of leukocytes, including monocytes, macrophages, eosinophils and basophils 40. Regarding functions, DC-SIGN plays a role in the anti-inflammatory activity of sialylated Fc by raising the threshold for myeloid cell activation, while CD23 mediates the selection of higher affinity antibodies for antigens by elevating FcγRIIb expression on B cells 29,41. In addition to the above proteins, Fc domain-interacting proteins also include (1) C1q, the classical promoter of the complement cascade; (2) neonatal crystallizable fragment receptor (FcRn), a recycling or transcytosis receptor for IgG; and (3) tripartite motif-containing 21 (TRIM21), a cytosolic receptor required for antibody-dependent pathogen constraint 42,43. Thus, the interaction between Fc and FcγRs can trigger diverse immune responses whose types are affected by the affinity of Fc.

### Glycosylation of IgG can affect the binding affinity between Fc and FcγRs

It has been reported that different subclasses of IgG demonstrate distinct binding affinity for FcγRs. For example, IgG1 and IgG3 exhibit the highest affinity for type I FcγRs, while IgG2 and IgG4 have lower affinities 31,32,44. In addition, Fc-associated glycoforms have been shown to profoundly impact these interactions. Although 10% of IgG3 molecules possess *O*-linked glycosylation at their hinge region, *N*-linked glycosylation can be found at Asn-297 of the CH2 domains of all other human IgG subclasses. The majority of these covalently attached *N*-glycans are complex-type biantennary structures containing a core heptasaccharide comprised of GlcNAc and mannose residues 45-47. Variable addition or removal of sugar moieties (e.g., fucose, galactose, bisecting GlcNAc, or sialic acid) gives rise to Fc diversity. As a result, over 36 distinct glycans have been found in healthy individuals' sera 48.

The presence of these glycans is critical for the flexibility of the Fc regions, which is a determinant for FcγR binding affinity, and for maintaining the CH2 domains at a conformation suitable for Fc-FcγR interactions 32. The affinity of core-fucosylated IgG1 for FcγRIIIa is 100-fold lower than that of the defucosylated version due to the steric hindrance between the Asn162-glycan of FcγRIIIa and the Asn297-linked carbohydrate of Fc chain A 49. On the one hand, the fucose residue prohibits the protrusion of FcγRIIIa Asn162-glycan into the central cavity formed by two CH2 domains. Therefore, the dynamics of the receptor binding site could negatively regulate the Fc-FcγR interaction 50,51. On the other hand, the reduction in the formation of hydrogen bonds between Fc-Gln295 and mannose 5 on receptor carbohydrates could also affect this interaction 49. In addition, sialylation of Fc reduces its affinity for type I FcγRs, whereas its affinity for DC-SIGN or CD23 increases. Upon sialylation, the CH2 domain of Fc adopts a flexible conformation suitable for binding to type II FcγRs, while the binding site for canonical FcγRs near the hinge-proximal surface is occluded 52,53. Overall, it is generally thought that IgG *N*-glycosylation profoundly affects immune responses and is involved in the occurrence and development of autoimmune disease.

## Sialylated IgG in autoimmune diseases

*N*-Acetylneuraminic acid (Neu5Ac), also called sialic acid, is attached to the galactose residue and constitutes the nonreducing end of IgG Fc *N*-glycans 54. The Neu5Ac-galactose linkage is an α2,6-linkage in humans and an α2,3-linkage in Chinese hamster ovary (CHO) cells 55. Although serum IgG contains less than 15% sialylated IgG, sialylation has been shown to be a vital player in mediating anti-inflammatory activities in autoimmunity 16.

### Sialylated IgG as a biomarker for diagnosis and monitoring

The IgG sialylation level changes during the development and progression of a wide variety of autoimmune diseases (Table 1) 54. Low levels of sialylated glycans in serum total IgG or autoantibodies have been reported in a number of autoimmune diseases, including SLE, granulomatosis with polyangiitis (GPA), Kawasaki disease, RA, and Crohn's disease 20, 21, 56-58. In 2015, Lauc *et al.* demonstrated that the levels of major sialylated glycans (FA2G1S1, FA2BG2S1, and FA2G2S2) in total IgG decreased in three SLE patient cohorts of different ethnicities (Latin American Mestizo, Trinidad and China) 56. IgG1 and IgG2 sialylation also decreased in GPA, as confirmed by liquid chromatography coupled with mass spectrometry 57. Su *et al.* observed a negative correlation between the serum levels of total sialylated IgG and RF autoantibodies in RA patients, suggesting that sialylation levels may be promising serum biomarkers for RA disease activity and clinical diagnosis 59.

Anti-beta-2-glycoprotein 1 (anti-β2GP1) antibodies are commonly found in patients with APS and are associated with increased thrombotic risk 60. However, anti-β2GP1 antibodies can be detected in 3% of asymptomatic healthy controls 61. The frequency of anti-β2GP1 antibodies in healthy children was 6.6%, according to an investigation by Avcin and coworkers enrolling 61 subjects 62. Therefore, biomarkers with higher specificity are needed to distinguish seropositive healthy individuals from asymptomatic patients. Fickentscher *et al.* found significantly decreased serum sialylation levels in APS patients and asymptomatic carriers compared with those in a healthy population, which indicated that hyposialylated anti-β2GP1 IgG could be a new measure to distinguish healthy and asymptomatic populations 19. Serologic markers, such as RF and ACPA autoantibodies, are often used in the diagnosis of RA 8. However, approximately 15-25% of RA patients are negative for either autoantibody. Utilizing an innovative platform based on TiO2-PGC chips, Wang and coworkers identified sulfated FA2G2S1 (termed SGm2), a sulfated sialylated glycan on serum IgG, as a biomarker for RF and ACPA-negative patients, with a high prediction accuracy of 88% 9. Thus, altered sialylation could provide a better way to close the diagnostic gap in asymptomatic individuals and to enhance diagnostic sensitivity in autoimmune diseases where autoantibodies are less easy to detect.

Anti-proteinase 3 (anti-PR3) antibody, which is correlated with the oxidative burst of neutrophils, is a diagnostic and pathogenic hallmark of GPA 63. Kemna *et al.* observed that the mean sialylation ratio of anti-PR3 antibodies not only decreased in GPA patients but was also inversely related to the Birmingham vasculitis activity score, indicating that the IgG sialylation level may be utilized to assess disease severity. Importantly, this reduced sialylation level was confirmed by an *in vitro* study showing that desialylated anti-PR3 antibodies could significantly increase the production of H_2_O_2_ by neutrophils, which contributes to GPA progression 64.

In addition to serving as a promising diagnostic biomarker complementing or even surpassing the present methods in some aspects, changes in sialylated IgG levels in serum could also have the potential to monitor therapeutic efficacy in autoimmune diseases. Gińdzieńska *et al.* found that the total serum IgG sialylation level was elevated after 12 months of methotrexate therapy in RA patients 65. Engdahl *et al.* observed increased sialylation levels of both total IgG and ACPA autoantibodies in postmenopausal women with RA after estrogen (E2) replacement therapy 66. In chronic inflammatory demyelinating polyneuropathy patients, sialylated IgG Fc was reported to be superior to other biomarkers for evaluating treatment response, such as polymorphism of the transient axonal glycoprotein-1 gene or expression of inhibitory FcγRIIb on B cells, due to its less time-consuming, easily accessible and reliable detection process 67.

In addition, sialylated IgG shows great promise for confirming remission, as well as predicting or confirming flares 68. The prognostic capacity of the total IgG sialylation profile preceding relapse has been pronounced by Kemna *et al*. 69. Total IgG1 sialylation in GPA patients started to decrease from the time of relapse, while it remained relatively unchanged in nonrelapsing patients, indicating that low sialyation of total IgG1 could predict disease relapse 69. In Guillain-Barré syndrome (GBS), it has been observed that patients with higher galactosylated and sialylated IgG1 and IgG2 levels require ventilator support less often and that they require less time to recover walking ability six months after receiving a high-dose IVIg treatment regimen 70. In contrast, patients with persistent low galactosylated and sialylated IgG1 and IgG2 levels had the most severe forms of GBS and showed poor response to IVIg treatment, suggesting that serum IgG Fc glycosylation in GBS is related to clinical recovery 70. Similarly, a persistent low level of serum sialylated IgG predicted IVIg resistance in patients with Kawasaki disease 20.

However, negative or inconsistent observations have been reported. For example, Wuhrer *et al.* found no significant changes in IgG Fc glycosylation in serum samples from multiple sclerosis patients compared to samples from healthy controls 71. Myrthe *et al.* observed that high serum Fc galactosylation and sialylation of anti-c autoantibodies positively correlated with disease severity (*P*=0.008 and *P*=0.039 respectively) in hemolytic disease of the fetus and newborn (HDFN). The increased galactosylation of anti-c is in line with studies showing a positive correlation between galactosylation and the binding of IgG1 to FcγRIIa and FcγRIIIa, ADCC activity and clinical outcome in fetal or neonatal alloimmune thrombocytopenia 72-74. However, as no major effect of sialylation on FcγR binding affinity has been reported in the literature, this positive correlation may not be direct and could be explained by significantly increased levels of galactosylation, as well as the close relationship between galactose and sialic acid as terminal sugars 75. Moreover, similar to other posttranslational modifications, IgG sialylation also has tissue specificity. Scherer *et al.* found low levels of sialylation in both total and ACPA IgG1 in RA patient sera, while decreased sialylation was found only in ACPA IgG1 in paired synovial fluid 76.

### Sialylated IgG as a therapeutic strategy

Sialylated IgG is a dynamic marker that has value for precision medicine, considering its close relationship with disease presence, progression, recurrence and response to therapeutics. Changes in the level of sialylation are also factors that shape the development of certain autoimmune diseases. Sialylated IgG-based therapeutics developed through glycoengineering present an exciting approach, as they can reset the threshold for immune cell activation 87,88. This strategy could correct rather than suppress the immune imbalance with minimal side effects and long-term efficacy 88. In the past ten years, preclinical and clinical progress has been made (Table 2), as we describe in detail below.

#### Therapeutic efficacy of sialylated IVIg

IVIg consists of pooled IgG from healthy donor plasma and is approved by the FDA for the treatment of acute or chronic inflammatory diseases. Although over 100 tons of IVIg is consumed worldwide every year, its high costs and finite supply limit its clinical applications 89-91. The immunomodulatory power of IVIg lies in its interference with multiple innate and adaptive cells. For example, IVIg induced autophagy in peripheral blood mononuclear cells, which restricted to monocytes, dendritic cells and M1 macrophages but not in M2 macrophages 92. Phagocytic capabilities of human monocyte-derived or splenic macrophages were also inhibited by IVIg 93,94. And IVIg activated Wnt-β-catenin pathway in human dendritic cells (DCs), while the transcription factor blocks inflammatory mediators and favored tolerogenic DCs 95. As for adaptive cellular immunity, IVIg expanded Tregs via induction of cyclooxygenase-2-dependent prostaglandin E2 in human DCs 96. Besides, mechanisms of IVIg activity have been subdivided into Fab- and Fc-dependent, considering IgG's function domains. Among them, sialic acid-enriched Fc has been demonstrated as an active component of high-dose IVIg with potent anti-inflammatory properties 39. In 2006, Kaneko and coworkers fractionated sialic acid-modified structures from IVIg through a *Sambucus nigra agglutinin* (SNA)-lectin affinity column. They found that 0.1 g/kg SNA IVIg could function as efficiently as 1 g/kg unfractionated IVIg 97. Recent evidence has also confirmed that sialylated IgG is indispensable for protecting mice from allergic bronchopulmonary aspergillosis, as desialylated IVIg fails to suppress Th2, Th17 and IgE responses 98. *In vitro*, sialylated IVIg inhibited complement activation in sera from patients with Guillain-Barré syndrome, Miller Fisher syndrome and multifocal motor neuropathy, while galactosylated, degalactosylated, or deglycosylated IVIg could not 99. Taking advantage of glycosyltransferase engineering, both α2,6 sialyltransferase (ST) recombinant Fc (rFc) and α2,3ST rFc were generated. Surprisingly, a one-thirtieth dosage of α2,6ST rFc reduced joint swelling to an extent similar to 1 g/kg IVIg, with increased IL-33 production and FcγRIIb upregulation in a mouse model of arthritis 22,28. Despite these benefits, however, minor sialylated IgG still lacks quantitative controls in the manufacturing process. In 2015, Nathaniel *et al.* prepared tetrasialylated IVIg (s4-IVIg) with minimal byproducts using a flow-through method. S4-IVIg showed at least 10-fold enhanced anti-inflammatory activities in mouse models of CIA, K/BxN arthritis, ITP, and epidermolysis bullosa acquisita, subsequently inhibiting the progression of the diseases 100. Of note, this process generating sialylated IgG products with consistent anti-inflammatory efficacy is suitable for clinical development. Furthermore, M254, the commercial derivative of s4-IVIg, is undergoing a randomized clinical trial (NCT03866577) 101. This trial is evaluating the safety, tolerability, pharmacokinetics, and pharmacodynamics of M254 in a total of 70 healthy volunteers and ITP patients. Additionally, the outcome of a head-to-head comparison between M254 and IVIg remains to be seen 102. In addition to the abovementioned glycoengineering strategies, Fiebiger and coworkers generated an IgG Fc variant with a point mutation at residue F241 (F241A), which mimics the conformation of sialylated IgG. Mechanistically, in contrast to the increased expression of FcγRIIb through α2,6ST rFc, F241A protected against the development of experimental autoimmune encephalomyelitis (EAE) in a mouse model, along with the expansion of Treg cells and the suppression of CD4^+^ effector T cells in the draining lymph nodes. Treg cell-depleted mice were devoid of F241 protection. Therefore, this point mutation F241A suppresses the pathogenic CD4^+^ T cell response by activating and expanding Treg cells 103.

However, it has to mention that the necessity of sialylated Fc has also met challenges in IVIg-mediated anti-inflammatory effects. Recent studies included Fc-sialylation-independent mechanisms of IVIg actions in the therapeutic collagen Ab-induced arthritis, EAE and ITP mouse model, as no difference in the therapeutic ability of IVIg was observed between sialic acid-enriched and -depleted groups 104-106. Additional studies from a human *in vitro* whole blood assay, found anti-inflammatory effects were most robust with Fab-sialylated fractions, rather than Fc-sialylated fragments 107. It is likely that more than one of the IVIg potential modes, together with pathogenetic heterogeneity from disease to disease, accounts for these differences.

Although most therapeutic attempts to enhance sialylation of both total IgG and autoantibodies are still in the preclinical stage, they have shown great potential to weaken and transform pathogenic antibodies and to reset the immune threshold. It is certain that additional drug candidates will emerge in the near future through advanced glycoengineering. It would be intriguing to unravel the broader indications and accurate usages of sialylated IVIg and autoantibodies, as well as the feasibility of their use in humans.

#### Therapeutic efficacy of sialylated endogenous IgG and pathogenic autoantibodies

In addition to exogenous IgG supplementation, studies have also been conducted to increase the sialylation of endogenous antibodies. For instance, collagen-induced arthritis (CIA) mice were orally fed the sialic acid precursor *N*-acetylmannosamine (ManNAc) by Ulrike *et al.*, which significantly increased serum IgG1 Fc sialylation. ManNAc-fed mice had a decreased CIA incidence, delayed arthritis onset and mitigated bone loss compared with mannose-fed or water-fed mice 108. This finding demonstrated a protective role of sialylated endogenous IgG against immune-mediated bone loss.

Autoantibodies are thought to be more crucial drivers in the pathogenesis of autoimmune diseases than total IgG 109. Compared with untreated antigen-induced arthritis (AIA)-IgG, the administration of sialylated (AIA) IgG before injecting methylated bovine serum albumin into BALB/c mouse knee joints could reduce arthritogenic activity and CIA severity 108. Consistently, sialylated anti-2,4,6-trinitrophenyl IgG1 could extend the survival of mice with nephrotoxic nephritis (NTN) beyond 18 days 110. By fusing human IgG1 Fc to ST6GAL1 and/or B4GALT1, a soluble form of the membrane proteins was engineered by Anthony *et al.* B4ST6^FC^ but not B4^Fc^ or ST6^Fc^ led to an increase in sialylation of pathogenic IgG in K/BxN-treated mouse paws and NTN-induced mouse kidneys compared to that in mice treated with PBS or IVIg. Mechanistically, the anti-inflammatory activities of B4ST6^Fc^ require the activation of DC-SIGN and STAT6 as well as the inhibitory FcγRIIb signaling pathway 87,111. Additionally, Ohmi and coworkers demonstrated that the observed anti-inflammatory effects were specific to citrullinated collagen type II (Col II) 78.

### Mechanisms of the anti-inflammatory effects of sialylated IgG in autoimmune diseases

#### ST6GAL1 is the key enzyme involved in the process and biosynthesis of sialylated IgG

**Figure 2:**
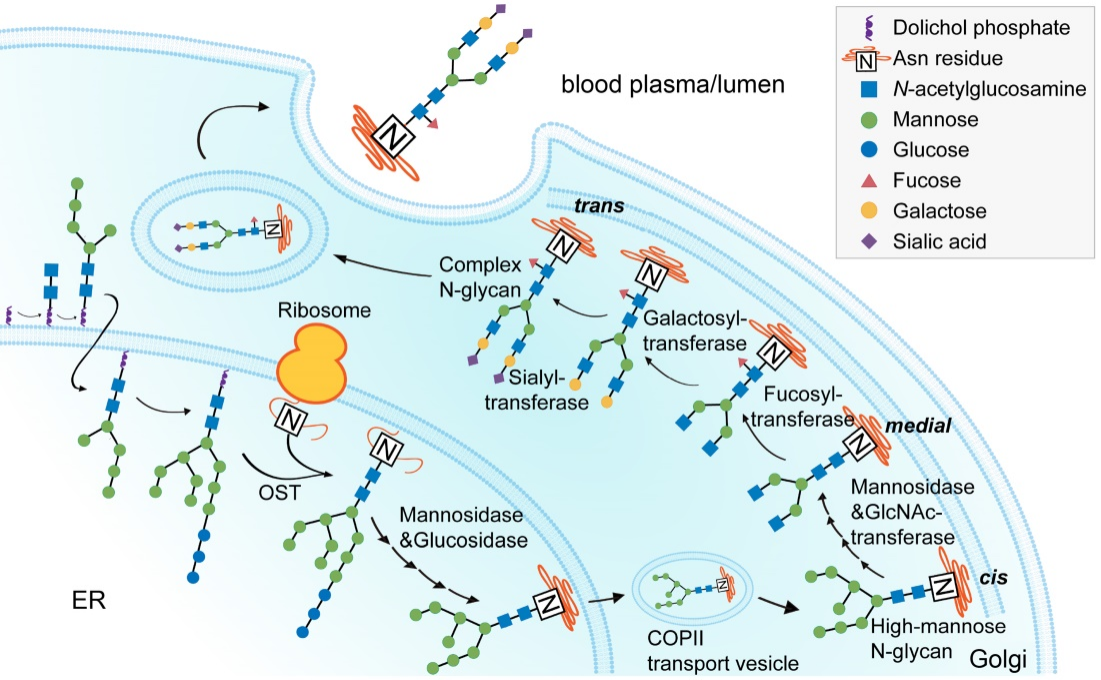
** Process of IgG *N*-glycan biosynthesis in the secretory pathway.** The process of *N*-glycosylation begins in the endoplasmic reticulum (ER) and ends in the Golgi. A lipid-linked precursor oligosaccharide is synthesized and transferred to the Asn residue in the ER, followed by initial trimming, transfer to the Golgi, modification with terminal sugar residues, and finally secreted into the lumen or blood plasma. OST, oligosaccharyltransferase.

The intracellular process of glyco1sylation begins in the endoplasmic reticulum and ends in the Golgi (Figure 2), during which sialylation is catalyzed by sialyltransferases 45,112. ST6GAL1 is the rate-limiting enzyme that transfers α2,6-linked sialic acids from a donor to the oligosaccharide chains of a glycoprotein 113,114. There are two forms of ST6GAL1: the secreted, soluble form and the membrane-bound form. The soluble form of ST6GAL1 can be produced by cells lining the central veins of the liver, with enzymatic activity to catalyze IgG sialylation in circulation, while the membrane-bound form of ST6GAL1 is widely expressed in B cells and typically found in the Golgi apparatus 115. The two forms of ST6GAL1 can both be altered by inflammatory challenges under autoimmune conditions. Hormone levels are associated with notable changes in this enzyme. In ovariectomized mice, E2 increased ST6GAL1 expression in plasmablasts as well as Fc sialylation of ovalbumin-specific IgG 66. In addition to hormones, aberrant blood factors have also been reported. In the CIA mouse model, activated T helper 17 (Th17) cells regulated ST6GAL1 expression on newly differentiated antibody-producing cells in an interleukin-21 (IL-21)- and IL-22-dependent manner, which subsequently changed the glycosylation profile and the activity of IgG produced by plasma cells.

This sequence of events clearly shows the correlation between the glycosylation machinery of B cells and inflammation initiation 116.

#### Mechanisms of the anti-inflammatory effects of sialylated IgG

To understand how sialylated IgG exerts anti-inflammatory effects in autoimmune diseases, two main cell subsets should be considered: myeloid cells and B cells.

**Figure 3:**
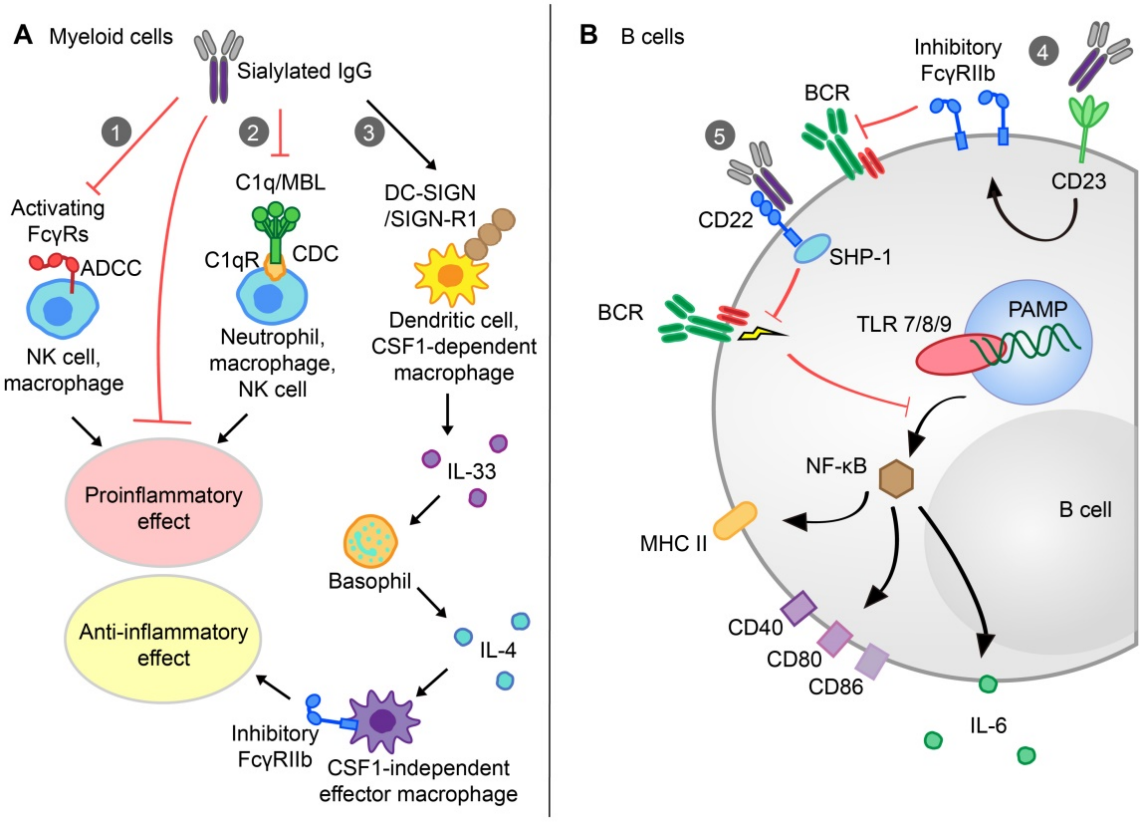
** The function and underlying mechanisms of sialylated IgG (s-IgG) in myeloid and B cells. A.** Upon IL-33 secretion by myeloid cells after s-IgG binding to DC-SIGN/SIGN-R1 (1), basophils produce IL-4, thus upregulating inhibitory FcγRIIb on CSF1-independent effector macrophages and resulting in increased anti-inflammatory effects. Additionally, CDC and ADCC activity decreases due to the reduced affinity of s-IgG for C1q (2) and activating FcγRs (3), respectively, which lead to a reduced proinflammatory effect.** B.** s-IgG can bind to CD23 (4) and increase the expression of inhibitory FcγRIIb on B cells. S-IgGs can also bind to CD22 (5) in an Fc-dependent manner and induce inhibitory SHP-1 recruitment, which inhibits BCR and TLR signaling pathways in B cells, further inhibiting the NF-κB signaling pathway, downregulating the expression of MHC II, CD40, CD80, and CD86 molecules and decreasing the expression of IL-6. ADCC, antibody-dependent cellular cytotoxicity; NK cell, natural killer cell; MBL, mannose-binding lectin; CDC, complement-dependent cytotoxicity; DC-SIGN, dendritic cell-specific ICAM-3-grabbing nonintegrin; SIGN-R1, specific ICAM-3-grabbing nonintegrin-receptor 1; IL, interleukin; CSF1, colony-stimulating factor 1; CD, cluster of differentiation; BCR, B cell receptor; SHP-1, SH2 domain-containing phosphatase 1; PAMP, pathogen-associated molecular pattern; MHC, major histocompatibility complex; TLR, toll-like receptor; NF-κB, nuclear factor-κB.

Several studies have demonstrated that a high level of IgG Fc sialylation was associated with decreased ADCC or complement-dependent cytotoxicity (CDC) activity due to the reduced affinity of human polyclonal IgG1 for FcγRIIIa or C1q (Figure 3A) 83,117. Compared with the asialylated G2F glycovariant, S2G2F IgG showed reduced affinity for the FcγRIIIa-V158 and F158 alleles. Thus, compared with administration of the core-fucosylated mAb in FcγR-humanized mice, S2G2F anti-mCD4 administration could prevent CD4^+^ T cell depletion in the blood 26. Researchers have proposed the requirement of sialylated IgG Fc with SIGN-R1 in models of inflammatory arthritis and skin blistering diseases under therapeutic conditions by using either mice deficient in SIGNR1 or SIGNR1-blocking Abs 118. The capability of its human ortholog DC-SIGN to replace SIGN-R1 in sialylated IVIg-mediated therapeutics effects has been verified in K/BxN arthritis models 41. However, the indispensability of these receptors has been challenged, as different groups have reported mouse SIGNR1 or its human ortholog DC-SIGN-independent protective effects of IVIg. For example, IVIg activity remained in SIGNR1-deficient mice or splenectomized C57BL/6 mice lacking SIGNR1-positive splenic marginal zone macrophages in ITP under therapeutic treatment conditions 118. Glycan array studies indicated that Fab fragments of IVIg were responsible for the binding between hypersialylated IgG and DC-SIGN 119. Moreover, Massoud* et al.* observed the interaction of sialylated IVIg with dendritic cell immunoreceptors (DCIRs) constructed on DCs, mediating Treg cell expansion and airway hyperresponsiveness attenuation 120. These differences may be attributed to the involvement of different immunoregulatory pathways between humans and mice, as well as the existence of disease-specific mechanisms for given clinical states.

After the engagement of human DC-SIGN with sialylated IgG, a Th2-dependent pathway was triggered, involving the production of IL-33 and the expansion of IL-4-produing basophils. Upon stimulation with IL-4, effector macrophages upregulated the expression of inhibitory FcγRIIb and increased the threshold of immune complex (IC)-mediated activation 41. Again, differences between model systems have been noted. Plasma IL-33 of rheumatic patients increased after infused with high-dose IVIg but was insufficient to mediate basophil expansion 121. Similarly, expansion of peripheral Treg cells, but not the up-regulation of plasma IL-33, was associated with clinical recovery following IVIg therapy in GBS patients 122. Recent evidence also showed that IVIg activated IL-3-primed human basophils and subsequent IL-4 secretion by directly interacting with surface-bound IgE Fab fragments independent of IL-33, FcγRIIb and C-type lection receptors 123. Overall, the action of Fc-sialylated IVIg does not involve a single mechanism. These board mechanisms need to be tested in distinct and specific models of autoimmune disorders.

In addition, Th17 cells also seem to be associated with downstream pathways. Bartsch *et al.* found that sialylated anti-Col II IgG1 antibodies significantly attenuated Col II-induced arthritis in FcγRIIb-deficient mice, with decreased accumulations of Th17 cells in the popliteal and brachial lymph nodes, in line with the *in vitro* finding that coculturing bone marrow-derived DCs from FcγRIIb-deficient mice with sialylated ICs inhibited secretion of IL-6, the critical cytokine for Th17 generation 110. However, additional studies are still required to identify the exact mechanism through which sialylated IgG impacts Th17 cells.

B cells can also be specifically modulated by sialylated IgG (Figure 3B). CD23 is a type II FcγR expressed on activated B cells. Wang *et al.* elaborated that after sialylated Fc glycans (sFc) bound to CD23, the inhibitory FcγRIIb on activated B cells was elevated, which resulted in B cells selecting higher affinity BCRs 29. CD22 is another inhibitory receptor expressed by B cells. Seite and colleagues observed that specific binding of sialylated IVIg to CD22 inhibited the BCR signaling pathway by downregulating tyrosine phosphorylation on numerous kinases 124. After treatment with sialylated IVIg, B cells became nonresponsive, with decreased antigen presentation ability, reduced expression of costimulatory molecules (MHC II, CD40, CD80, and CD86) and decreased secretion of inflammatory cytokines (IL-6/IL-10).

## Other glycans

In addition to sialylation, IgG fucosylation or galactosylation has been widely documented across various autoimmune diseases 15. Glycosyltransferases conjugate sugar moieties to nascent proteins and lipids, thus generating a wide variety of glycans 125. Meta-analysis of genome-wide association studies identified four glycosyltransferase (ST6GAL1, B4GALT1, FUT8 and MGAT3)-coding loci that are strongly associated with autoimmune conditions 126.

### Galactose

Galactose, which links to GlcNAc, is a widely studied glycan moiety in autoimmunity 54. Since the 1980s, the majority of galactosylation-related studies have focused on the impact and correlation of decreased IgG *N*-linked galactosylation in RA. A high percentage of IgG G0 (no galactose) glycoforms was positively correlated with the RA state and severity, which reverted to normal levels upon MTX treatment or pregnancy-induced remission 65,127-129. This G0 change occurred preferentially in ACPA, as SF ACPA-IgG1 but not with total SF IgG1 was highly agalactosylated 76,129. Importantly, ACPA-IgG1 Fc exhibited decreased galactosylation three months prior to RA diagnosis, but this was not observed in undifferentiated arthritis populations 21. Overall, the level of galactosylation could be informative with respect to pathogenetically relevant inflammatory processes pre-RA and may be of value in early intervention. In addition, hypogalactosylation of both total IgG and specific autoantibodies has also been reported in spondyloarthropathy, SLE, ANCA-associated systemic vasculitis, inflammatory bowel disease, myasthenia gravis and primary Sjögren's syndrome 3,56,81,130-132. Through detailed studies of the Fc portion, the observation of galactosylation defects was expanded to GPA and autoimmune hemolytic anemia 69,133.

Despite the alterations in galactosylation patterns in a variety of autoimmune diseases, inconsistencies have also been observed among the immune responses triggered by galactosylated IgG. Early research discovered that IgG with G0F glycans exhibited a high affinity for FcγRIII, contributing to antibody-mediated inflammation 74,134. However, the binding rate of hypofucosylated and hypergalactosylated IgG1 to FcγRIIIa increased 40-fold, while hypofucosylated IgG1 showed a 17-fold affinity increase, resulting in enhanced NK cell-mediated ADCC against red blood cells 135. Hypergalactosylated IgG1 ICs facilitated the association of FcγIIb with dectin-1, leading to the blockage of C5a-dependent inflammatory responses *in vitro* and* in vivo* 136. Moreover, hypergalactosylated IgG1 showed increased binding to C1q, with the de novo ability to lyse Burkitt lymphoma-derived Raji cells 135,137. Further studies are needed to resolve these inconsistencies and to assess the roles of galactosylation in therapy.

### Fucose

In the serum IgG pool, nearly 90% of Fc-tail glycoforms are core fucosylated. Serum Fc fucosylation is relatively stable throughout life and in the majority of autoimmune conditions 54. Some studies have reported skewed fucosylation found in fetal and neonatal alloimmune thrombocytopenia and HDFN apart from SLE 27,56,73,75. Lower core fucosylation was positively correlated with clinical severity, a feature found for both anti-human platelet antigen (HPA)-1a and anti-D IgG1 antibodies 73,75,138,139. Anti-c and anti-E antibodies were undetectable or showed far lower levels, while low fucosylation was observed for anti-D antibodies 75. Importantly, decreased fucosylation of IgG increased its affinity for human FcγRIIIa/IIIb, in line with a 50-fold higher ADCC activity of less-fucosylated humanized mAbs through more flexible glycan-glycan interactions between the Fc domain and FcγRIII 28,140. Such observations led to the development of defucosylated or hypofucosylated antibodies, especially for antitumor therapy, where cytotoxicity is needed 141. For example, mogamulizumab, a defucosylated mAb targeting CC‑chemokine receptor 4, broadens the spectrum of treatment options for adult T-cell leukemia/lymphoma and cutaneous T-cell lymphoma 142.

## IgG sialylation glycoengineering approaches

In 2006, Jeffrey Ravetch's team reported that the anti-inflammatory activity of high-dose IVIg depended on terminal α2,6-sialylated IgG 18,97. Then, recombinant glycosylated IgG with tailored effector function was demonstrated to be a promising therapeutic strategy 111. Recent progress in glycoengineering has provided strategies to produce glycan-defined homogeneous antibodies. They include an *in vivo* genetic method focusing on *N*-glycan biosynthetic system manipulation and *in vitro* metabolic remodeling via glycosyltransferase or endoglycosidase activity (Table 3). The following sections will discuss recent advances in these areas 143.

### Genetic glycoengineering

Host cell engineering through genetically modifying mediators in the intracellular glycan biosynthetic pathway has been the most widely used approach 55. Various permanent edits in glycosylation can be achieved via genome mutation and knockout, as well as upregulation or downregulation of glycosylation enzymes 144. α2,6 ST and α2,3 ST, which can catalyze the addition of monosaccharide residues, are the most frequently studied glycosylation enzymes. Raymond and coworkers coexpressed ST6 and β1,4-galactosyltransferase 1 in CHO cells to produce F241A mutant IgG1 with 85% α2,6-linked sialic acid 145. Other attempts include transfection of cytidine monophosphate-sialic acid transporter (CMP-SAT) to increase precursor sugar levels or inhibit sialidase to eliminate terminal carbohydrate hydrolysis. Likewise, advances in genome editing technologies, such as CRISPR/Cas9, have made it more feasible to produce pure or enriched α2,6-sialylated mAbs 146,147. Blundell *et al.* introduced new *N*-glycosylation sites to hexa-Fcs, which enhanced its sialylated glycan composition up to 75% 148.

However, the glycoengineering output quality and efficiency may deviate from the desired level due to the restrictions of mammalian host cells. CHO cells are the preferred *in vitro* expression platforms, allowing for high levels of human-like posttranslational modifications of proteins. However, *N*-glycolylneuraminic acid (Neu5Gc), a nonhuman sialic acid, can be synthesized by CHO cells to occupy the same epitopes as Neu5Ac. Neu5Gc-glycoprotein injection gives rise to an abnormal anti-Neu5Gc antibody response, and the combination of Neu5Gc-containing epitopes and anti-Neu5Gc antibodies may exacerbate colorectal cancer and atherosclerosis 149-151. Variability in cell culture parameters could also affect glycosylated protein expression levels. Lower cell culture temperature was reported to be associated with an increase in IgG3 sialylation 152. Furthermore, glycoproteins are produced as glycoformic mixtures that have the same protein backbone but differ in the attached sugar chains. It is still difficult to isolate pure glycoproteins using current chromatographic techniques 153.

### Glycosyltransferase glycoengineering

Parallel to *in vivo* genetic approaches, *in vitro* glycan chain extension by glycosyltransferase has paved the way for producing homogenous glycoproteins with structurally defined oligosaccharides 143,154. A one-pot system composed of two enzymes (ST6GAL1 and B4GAL1) as well as the corresponding substrate molecules (uridine-5'-diphospho-galactose and CMP-sialic acid) was used to generate tetra-Fc-sialylated IVIg. Washburn and coworkers maximized enzymatic incorporation while minimizing byproducts by optimizing the ratio of CMP-sialic acid to ST6GAL1 100. Likewise, 74% sialylated human IgG has been generated by Dekkers utilizing recombinant ST6GAL1 and CMP-sialic acid substrates 155. Glycosyltransferase-based strategies have made it much simpler to acquire defined glycoproteins quickly. Despite these advances, the obtained glycoforms lack diversity due to the limited number of commonly used glycosyltransferases. Parameters, including enzymatic substrate specificity and favorable catalytic temperature, should be considered in next-generation glycosyltransferase glycoengineering 156. Another difficulty in acquiring expensive sugar nucleotides or sugar phosphate as active donor substrates limits its application in large-scale manufacturing.

### Chemoenzymatic glycoengineering

While the aforementioned approaches are impeded by the limited quality and diversity of glycoforms, chemoenzymatic glycoengineering, remodeling based on endoglycosidase (ENGase) for glycans and the chemical synthesis of highly active glycan oxazolines, has attracted considerable attention since 2008 143,153. Briefly, chemoenzymatic glycoengineering consists of two enzymatic steps: deglycosylation to trim heterogeneous IgG by ENGase, leaving the innermost GlcNAc residues, and subsequent addition of a predefined oligosaccharide substrate to the GlcNAc peptide by glycosynthase mutants 143,157. Both purified mAbs and antibody mixtures, such as IVIg, have been successfully modified using chemoenzymatic glycoengineering.

Contrary to the narrow substrate specificity of other endoglycosidases, Endo-S secreted by *Streptococcus pyogenes* shows high specificity for IgG and can act only on biantennary complex glycans 158,159. The pair of Endo-S and its mutant (Endo-S-D233Q) showed great efficiency in generating bisialylated mAbs, including rituximab and trastuzumab 25,160,161. A glycosylase mutant (Endo-S2-D184M) from Endo-S2 was developed by Wang *et al.* 162,163, with improved efficiency of transglycosylation. Additionally, Endo-S2 can cleave all three types of Fc *N*-glycans, including hybrid, high mannose and complex types 164-166. Furthermore, Endo-S2-processed rituximab with a homogeneous terminal α2,6-sialylation showed clearly reduced affinity for FcγRIIIaV158 compared to that of asialylated antibodies 26. Utilizing Endo-S and Endo-S-D233Q, Wang and coworkers successfully purified minor α2,6-sialic acid from IVIg, increasing its purity from less than 10% to more than 90%. Moreover, the *N*-glycans on the Fab domains remained intact, which was confirmed by fluorescent HPLC profiles 167.

Chemoenzymatic glycoengineering is a versatile approach to optimize sizable homogenous IgG glycoforms. Its preparation time is relatively short, with 5 to 8 days to prepare glycan substrates and 3 to 4 days to engineer IgG glycosylation 168. However, the synthesis and purification of large quantities of oligosaccharide substrates are still in their infancy 169,170. Recent developments, such as the production of Man3GlcNAc oxazoline from egg yolks and Man9GlcNAc2 from soybean flour, have shown progress 171,172.

To summarize, glycoengineering will deepen our understanding of antibody glycosylation and facilitate the production of therapeutics modulating IgG sialylation.

## Conclusions and perspectives

The ultimate goal of ongoing research on autoimmune diseases is to improve diagnostic accuracy and specifically attenuate pathogenic self-reactive components while maintaining normal immune defense and surveillance 7,88. Great attention has been paid to IgG Fc sialylation, the alterations of which have been observed in a wide range of immune disorders 45. These changes in sialic acid can be used as biomarkers to facilitate diagnosis and prognosis, to monitor disease progression and to evaluate therapeutic efficacy. Furthermore, developments in glycoengineering have ushered in a series of sialylated IgG-based therapeutics.

The glycome is affected by genetic and environmental factors, and changes have been associated with the development of inflammation. Prior research, therefore, focused mainly on the cross-sectional and longitudinal associations between the biomarkers of autoimmune diseases, disease activity, therapeutic efficacy and sialylated IgG. However, the interactions between sialylated IgG and immune cells as well as the underlying mechanisms of the regulation of sialylated IgG on immune responses in autoimmune disease have not been fully elucidated and need further investigation. Moreover, as rheumatic diseases are often systemic, abnormal immune tolerance can lead to multimorbidity 173. Interestingly, links between cardiovascular or metabolic disease and IgG sialylation are emerging: in high-fat diet-fed mice, ManNAc restored IgG sialylation and prevented obesity-related insulin resistance, as well as obesity-induced hypertension 174,175. In this respect, it would be helpful to elucidate additional roles of sialylated IgG in rheumatic patients with multimorbidity. In addition, the latest literature has indicated that these sialylated IgG therapeutic interventions may also attenuate IgE-mediated allergic reactions by upregulating inhibitory FcγRIIb on immune cells 176. Their application range may be expanded to allergies, infections, and even tumors, while their detailed mechanisms are still under study 177.

Although advancements in glycoscience have led to progress in glycan-based therapeutics and continued to provide exciting prospects, further studies are expected to achieve commercially available glycoengineered antibodies and site-specific antibody-drug conjugation 178. With the advancement of glycobiology and glycomedicine, we anticipate that in the near future, glycan analysis will become integral to the diagnosis, prognosis and management of autoimmune diseases.

## Figures and Tables

**Table 1:** Clinical significance of sialylated IgG in human autoimmune diseases

Disease	Serum levels of sialylated IgG (s-IgG)	Clinical significance	Ref.
Rheumatoid arthritis (RA)	Total s-IgG↓ RF s-IgG↓ ACPA s-IgG↓	**Enhanced diagnostic sensitivity:** Sulfated FA2G2S1 was identified as a biomarker to distinguish RA patients from the ACPA and RF-negative ones with high sensitivity **Therapeutic response:** Serum s-IgG was significantly elevated after methotrexate therapy in RA patients; Negative correlations of s-IgG were observed between DAS 28 and *Sambucus nigra* before and after therapy.	9,59,65,77,78
Juvenile idiopathic arthritis (JIA)	Total s-IgG↓	Total IgG sialylation was reduced in JIA patients' sera compared with that of healthy controls	79
Systemic lupus erythematosus (SLE)	Total s-IgG↓ Anti-histone s-IgG↓	Three major sialylated glycans in total IgG were decreased in SLE patients of African Caribbean populations, Latin Americans of Mestizo ethnicity and Han Chinese populations **Disease intensity:** Monosialylated glycans in total s-IgG were negatively associated with symptom profiles of SLE patients in African Caribbean and Latin American cohorts.	56,80
Anti-phospholipid syndrome (APS)	Anti-β2GP1 s-IgG↓	**Disease activity:** A significant negative correlation was observed between BVAS score and the sialylation ratio of PR3-ANCA. **Closing diagnostic gaps-asymptomatic individuals:** Sera from healthy children showed the highest *Sambucus nigra*/anti-β2GP1 s-IgG ratio. The ratio decreased from aaPL to SLE + aPL to SAPS to PAPS.	19,64
Granulomatosis with polyangiitis (GPA)	Total s-IgG↓ PR3-ANCA s-IgG1↓	Total s-IgG1 and s-IgG2 were reduced in GPA patients compared to healthy controls **Enhanced diagnostic specificity:** PR3-ANCA s-IgG was used to determine GPA activity with higher specificity and sensitivity than nonsialylated IgG. **Relapse:** Patients with low total s-IgG1 were highly prone to relapse after an ANCA increases. The degree of total s-IgG1 Fc was found to differentiate relapsing patients from nonrelapsing ones with high sensitivity and specificity.	57,64,69
Kawasaki disease (KD)	Total s-IgG↓	**Therapeutic response:** IVIg-resistant KD patients had lower levels of total s-IgG than IVIg-responsive KD patients at both pretreatment and one-year time points	20
Crohn's disease (CD)	Total s-IgG↓	The proportion of total s-IgG was significantly decreased in CD patients	58,81
Fetal and neonatal alloimmune thrombocytopenia (FNAIT)	Anti-HPA-1a s-IgG1↑	Anti-HPA-1a s-IgG1 increased up to 30% compared to total IgG sialylation of less than 10% in FNAIT patients.	73
Hemolytic disease of the fetus and newborn (HDFN)	Anti-D s-IgG↑ Anti-c s-IgG1↑	**Disease activity:** High Fc sialylation of anti-c was correlated with HDFN disease severity.	75,82
Chronic inflammatory demyelinating polyneuropathy (CIDP)	Total s-IgG Fc↓	s-IgG Fc was reduced in CIDP patients **Therapeutic response:** Reduction in clinical disease severity scores upon IVIg therapy was significantly associated with an induction of s-IgG Fc.	70,83
Guillain-Barré syndrome (GBS)	s-IgG2↓	**Therapeutic response:** IVIg therapy resulted in increased s-IgG1 and s-IgG2 in GBS patients A higher level of s-IgG1 and IgG2 Fc was associated with reduced disease severity and improved outcomes of GBS patients after IVIg therapy.	70,84
Alzheimer's disease (AD)	s-IgG1↓	S-IgG1 (FA2G2S1) was reduced in AD patients compared to patients with SMCI	85
Parkinson's disease (PD)	Total s-IgG↓	s-IgG sialylation decreased in PD patients **Disease activity:** FG2S1 was negatively associated with case status, with high sensitivity and specificity.	86

ACPA, anti-citrullinated protein antibody; anti-βGP1, anti-beta-2-glycoprotein 1; BVAS, Birmingham vasculitis activity score; PR3-ANCA, anti-neutrophilic cytoplasmic autoantibodies targeting proteinase 3; aPL, antiphospholipid antibody; aaPL, asymptomatic carriers of aPL; SLE + aaPL, patients with SLE without symptoms of APS harboring circulating aPL; SAPS, patients with APS and SLE as an underlying disease; PAPS, patients with primary APS; IVIg, intravenous immunoglobulin; HPA, human platelet antigen; anti-D, anti-rhesus D (RhD); SMCI, stable mild cognitive impairment.

**Table 2:** Therapeutic effect of IgG sialylation in experimental models of autoimmune diseases

Sialylated IgG	Method	Autoimmune disease	Outcome *in vivo*	Mechanism	Ref.
SNA-enriched IVIg	Fractionate IVIg on an SNA-lectin affinity column	RA	The reduction in clinical scores of arthritis was enhanced	Anti-inflammatory activity was enhanced through increased expression of inhibitory FcγRIIb on effector macrophages	99
α2,6 ST IVIg or rFc	Treat Fc with α2,3/6 sialidase, β1,4 GT and α2,3 or α2,6 ST in turn	RA	The reduction in clinical scores of arthritis was enhanced	Sialylation of Fc altered the binding affinity to FcγR, leading to a reduced ratio of binding to activating over inhibitory FcγRs	22
Sialylated 6A6-IgG2b		ITP	Platelet counts were significantly increased *in vivo*	Cytotoxicity of 6A6-IgG2b-mediated platelet consumption was reduced	
Sialylated AIA-IgG	Incubate IgG with CMP-sialic acid and α2,6ST	RA	Sialylated AIA-IgG showed excellent efficacy in preventing arthritis progression	Osteoclast differentiation and bone loss were prevented	108
Sialylated anti-Col II IgG1	Incubate IgG1 with β1,4 GT and α2,6 ST in the presence of UDP-galactose and CMP-sialic acid	RA	Sialylated Col II-reactive IgG autoAbs reduced the mean clinical score of arthritis symptoms	Activation of inflammatory DC and subsequent Th17 cell differentiation were inhibited and production of proinflammatory cytokines, such as IL-6 and IL-17, was reduced	110
Sialylated anti-TNP IgG1		NTN	Sialylated anti-TNP IgG1 in ICs reduced nephritis-induced mortality		
Sialylated anti-Col II antibody	Transfect *mB4galt1* and *mSt6gal1* cDNAs into anti-Col II lgG1 hybridomas	RA	Sialylated anti-Col II IgG antibodies prevented the development and progression of CAIA	Sialylated antibody treatment significantly increased the sialylation levels of anti-Col II IgG during an antigen-specific event, thus inducing regulatory activity	78
Sialylated autoantibodies	Generate recombinant glycosyltransferase enzymes by fusing human lgG1 Fc to ST6GAL1 and B4GALT1	RA	Elevated levels of sialylated autoantibodies induced by B4ST6^Fc^ reduced joint inflammation	B4ST6^Fc^ converted pathogenic IgG into anti-inflammatory IgG	111
		NTN	BUN level and kidney damage were reduced, and survival rate was increased		
s4 IVIg	Incubate recombinant human IgG1 Fc with β1,4 GT and α2,6 ST in the presence of UDP-galactose and CMP-sialic acid	RA	Enhanced potency when treated prophylactically compared to that of conventional IVIg	Neutrophil infiltration in ankle joints was inhibited	100
		ITP	Platelet levels were restored with enhanced efficacy		
		EBA	Therapeutic effect was enhanced when dosed prophylactically	Recruitment of inflammatory effector cells was inhibited and skin inflammation was reduced	
M254 (commercialized s4-IVIg)	Same as above	ITP	An ongoing randomized clinical trial (NCT03866577) is enrolling patients with ITP, expected to be completed in 2021		102

SNA, *Sambucus nigra* agglutinin; IVIg, intravenous immunoglobulin; RA, rheumatoid arthritis; rFc, recombinant Fc; ST, sialyltransferase; GT, galactosyltransferase; ITP, idiopathic thrombocytopenic purpura; CMP, cytidine monophosphate; UDP, uridine 5'-diphosphate; AIA, antigen-induced arthritis; Col II, collagen type II; TNP, 2,4,6-trinitrophenyl; IC, immune complex; IL, interleukin; Th, T helper; BUN, blood urea nitrogen; NTN, nephron toxic nephritis; EBA, epidermolysis bullosa acquisita.

**Table 3:** Comparison of glycoengineering methods

	Genetic glycoengineering	Glycosyltransferase glycoengineering	Chemoenzymatic glycoengineering
Strategies	Modify intracellular glycosylation pathways and enzymes via genetic engineering.	Extend monosaccharide residues by glycosyltransferases *in vitro.*	Modify sugar chains by endoglycosidases and their mutants, together with chemically synthesized active glycan oxazolines.
Methods	Remold sialyltransferases; increase CMP-Neu5Ac-associated enzymes or transporters; inhibit or eliminate sialidases; introduce new *N*-glycosylation sites.	Construct one-pot system with monosaccharide precursors and glycosyltransferases.	Deglycosylate IgG by an ENGase, prepare oxazoline derivatives as sugars donors via chemical methods, and transglycosylate oxazoline donor to glycoprotein.
Pros	Versatility	Simplicity and relatively purified products.	Simplicity; relatively purified and unlimited products.
Cons	Low efficiency and hybrid glyco-products	Limited glyco-products; difficulty and high cost of active glycan substrates.	Unavoidable hydrolytic activity of ENGase mutant; difficult to achieve oligosaccharide substrates in a large scale.

CMP, cytidine monophosphate; Neu5Ac,* N*-acetylneuraminic acid; ENGase, endoglycosidase.

**Table 4:** Overview of the glycan structures described in this review. For all glycoforms, blue square: *N*-acetylglucosamine, green circle: mannose, yellow circle: galactose, red triangle: fucose, purple diamond: *N*-acetylneuraminic acid. Glycan compositions are depicted with hexose (H), *N*-acetylhexosamine (N), deoxyhexose (F), and *N*-acetylneuraminic acid (S). Short names are given in terms of the diantennary (A2), galactosylated (G), sialylated (S), bisecting β (1,4) GlcNAc (B) and α (1,6) fucose (F) glycan attached to core GlcNAc.

Glycan structure	Compound name	Glycan short name
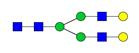	H5N4-IgG	A2G2
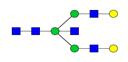	H5N5-IgG	A2BG2
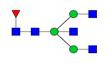	H3N5F1-IgG	FA2B
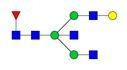	H4N5F1-IgG	FA2BG1
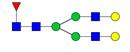	H5N4F1-IgG	FA2G2
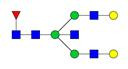	H5N5F1-IgG	FA2BG2
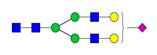	H5N4S1-IgG	A2G2S
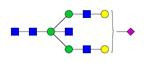	H5N5S1-IgG	A2BG2S
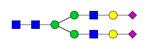	H4N5S2-IgG	A2G2S2
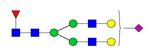	H5N4F1S1-IgG	FA2G2S
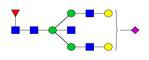	H5N5F1S1-IgG	FA2BG2S
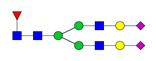	H5N4F1S2-IgG	FA2G2S2

## Abbreviations

IgG: immunoglobulin G
Fc: crystallizable fragment
FcγR: Fc gamma receptor
RA: rheumatoid arthritis
RF: rheumatoid factor
ACPA: anti-citrullinated protein antibody
mAbs: monoclonal antibodies
TNF: tumor necrosis factor
DC: dendritic cell
PML: progressive multifocal leukoencephalopathy
SLE: systemic lupus erythematosus
APS: antiphospholipid syndrome
IVIg: intravenous immunoglobulin
ITP: immune thrombocytopenic purpura
CCR: C-C chemokine receptor 4
FDA: Food and Drug Administration
NK: natural killer
BCR: B cell receptor
ADCC: antibody-dependent cellular cytotoxicity
Fab: antigen-binding fragment
ADCP: antibody-dependent cellular phagocytosis
DC-SIGN: Dendritic cell-specific ICAM-3-grabbing nonintegrin
FcRn: neonatal crystallizable fragment receptor
TRIM21: tripartite motif-containing 21
CH: constant heavy
CHO: Chinese hamster ovary
GPA: granulomatosis with polyangiitis
GBS: Guillain-Barré syndrome
HDFN: hemolytic disease of the fetus and newborn
FNAIT: Fetal and neonatal alloimmune thrombocytopenia
JIA: Juvenile idiopathic arthritis
CIDP: Chronic inflammatory demyelinating polyneuropathy
AD: Alzheimer's disease
PD: Parkinson's disease
KD: Kawasaki disease
CD: Crohn's disease
CIA: collagen-induced arthritis
AIA: antigen-induced arthritis
NTN: nephrotoxic nephritis
SNA: Sambucus nigra agglutinin
IL: interleukin
Th: T helper
CDC: complement-dependent cytotoxicity
IC: immune complex
ANCA: anti-neutrophilic cytoplasmic autoantibodies
ST: sialyltransferase
GT: galactosyltransferase
